# Human peptidergic nociceptive sensory neurons generated from human epidermal neural crest stem cells (hEPI-NCSC)

**DOI:** 10.1371/journal.pone.0199996

**Published:** 2018-06-28

**Authors:** Rachel Wilson, Afsara A. Ahmmed, Alistair Poll, Motoharu Sakaue, Alex Laude, Maya Sieber-Blum

**Affiliations:** 1 Institute of Genetic Medicine, Centre for Life, Newcastle University, Newcastle upon Tyne, United Kingdom; 2 School of Biology, Newcastle University, Newcastle upon Tyne, United Kingdom; 3 The Bio-Imaging Unit, Medical School, Newcastle University, Newcastle upon Tyne, United Kingdom; University of Colorado Boulder, UNITED STATES

## Abstract

Here we provide new technology for generating human peptidergic nociceptive sensory neurons in a straightforward and efficient way. The cellular source, human epidermal neural crest stem cells (hEPI-NCSC), consists of multipotent somatic stem cells that reside in the bulge of hair follicles. hEPI-NCSC and primary sensory neurons have a common origin, the embryonic neural crest. For directed differentiation, hEPI-NCSC were exposed to pertinent growth factors and small molecules in order to modulate master signalling networks involved in differentiation of neural crest cells into postmitotic peptidergic sensory neurons during embryonic development. The neuronal populations were homogenous in regard to antibody marker expression. Cells were immunoreactive for essential master regulatory genes, including NGN1/2, SOX10, and BRN3a among others, and for the pain-mediating genes substance P (SP), calcitonin gene related protein (CGRP) and the TRPV1 channel. Approximately 30% of total cells responded to capsaicin, indicating that they expressed an active TRPV1 channel. In summary, hEPI-NCSC are a biologically relevant and easily available source of somatic stem cells for generating human peptidergic nociceptive neurons without the need for genetic manipulation and cell purification. As no analgesics exist that specifically target TRPV1, a ready supply of high-quality human peptidergic nociceptive sensory neurons could open the way for new approaches, in a biologically relevant cellular context, to drug discovery and patient-specific disease modelling that is aimed at pain control, and as such is highly desirable.

## Background

Many serious diseases, including cancer, heart disease, diabetes, AIDS and arthritis, are often associated with unmitigated pain. Despite major advances in our understanding of the molecular mechanisms underlying pain and even though the potential drug targets identified by the pharmaceutical industry have increased dramatically, there are still only a few analgesic drug classes, primarily opioids and aspirin-like drugs, all of which have safety issues [1].

Given this situation, it would be extremely useful for the testing of potential new drugs and for the elucidation of the molecular mechanisms that result in the perception of pain for there to be readily available populations of human neurons that convey pain. Because neural crest cells give rise to nociceptive peptidergic sensory neurons, the overall goal of the present study was to determine whether hEPI-NCSC could be differentiated efficiently into peptidergic nociceptive neurons that respond to capsaicin.

Some diseases, including diabetic neuropathy, migraine, asthma, inflammatory bowel disease, interstitial cystitis, chronic cough, and osteoarthritis as well as cancer-related pain have a significant neurogenic inflammatory component [2, 3]. This type of pain is conveyed by unmyelinated sensory neurons (C-fibres) and a subset of sensory neurons with thinly myelinated axons (Aδ fibres) that are sensitive to capsaicin, the pungent substance in chili peppers. Capsaicin specifically activates TRPV1 (transient receptor potential vanilloid subfamily, member 1) in these nociceptive neurons. Activation of TRPV1 causes release of the neuropeptides substance P (SP) and calcitonin gene-regulated peptide (CGRP). These neuropeptides transduce pain and are also involved in triggering the inflammatory response, which plays a central role in neurogenic pain [4].^.^ In addition, the capsaicin receptor TRPV1 can become up-regulated in hyperalgesia due to peripheral nerve injury and in some cases of diabetic neuropathy [2,3,5–9]. TRPV1 is a major target for pain relief medication because it is thought that endogenous agonists may play a major role in certain pain conditions. A number of small-molecule TRPV1 antagonists are undergoing clinical trials and have been found to be useful [3] with the potential complication, however, that redundant pain pathways may exist [10]. Conversely, due to the rapid desensitization of TRPV1, therapies using TRPV1 agonists are of interest also.

The central role of TRPV1 in the transduction of pain and in initiating the neurogenic inflammatory response is well established [3]. The lack of effective drugs for the above conditions highlights the need for further investigation into the therapeutic potential of TRPV1 antagonists. For these reasons, the availability of human peptidergic nociceptive neurons is highly desirable. Neural crest cell-derived sensory neurons have the advantage over available cell lines that are unrelated to sensory neurons that it will be possible to study TRPV1 receptor action within the correct cellular context.

The experimental approaches used in the present study are based on the available literature. There is a large body of literature on nociceptive peptidergic neurons in rodents and in rodent development. In contrast little is known about the mechanisms that underlie human peptidergic nociceptive sensory neuron development, and observations made in rodents are not always translatable to humans. Conversely, many master signalling pathways are conserved during evolution and are therefore likely applicable to human peptidergic nociceptive neurons also. NGN1/2 are master regulatory genes essential for sensory neuron differentiation. SOX10 and canonical WNT signalling regulate expression of NGN1 and NGN2 [5, 11, 12], whereas Notch/Delta signalling inhibits NGN1 and NGN2 expression [6, 13]. There is also a positive feedback loop in which NGN2 activates Notch/Delta signalling [13]. NGN1/2 in turn regulate BRN3a expression, as does NT-3 [14, 15], among other signalling pathways. BRN3a is a specific pan-sensory neuron marker that is essential for further differentiation. BRN3a regulates RUNX1, another essential regulatory gene [16]. RUNX1 is also up-regulated by NGF [17] and ISL1 [16, 18]. In turn, RUNX1 up-regulates TRKA [16]. NT-3 and NGF can also be involved in TRKA expression [19, 20]. Conversely, RUNX1 can also inhibit TRKA and CGRP [21, 22]. NGN2, but not NGN1, expression is essential to guide neural crest cells towards the sensory neuron cell fate and it is up-regulated by SHH and WNT signalling [5]. Signalling by the evolutionary conserved master-gene, SHH, is required for dorsal root ganglion development and patterning and it enhances BRN3a expression in zebrafish [23] and neuron patterning in the chick DRG [24]. Sensory neurons are formed in two waves; large diameter sensory neurons are generated first and are followed by small and medium diameter sensory neurons [5, 25, 26]. Later, NGN1/2 and subsequently RUNX1 and TRKA are expressed in future nociceptive sensory neurons [5]. In a third wave of neurogenesis, boundary cap cells contribute mainly to RUNX1+/TRKA+ sensory neurons. Peptidergic nociceptive neurons lose RUNX1 expression, which is thought to be achieved by HGF (hepatocyte growth factor, scatter factor) via its receptor MET [5, 22]. In peptidergic nociceptive sensory neurons, TRKA remains expressed. In contrast, in non-peptidergic nociceptive neurons, TRKA is down-regulated postnatally, RET is expressed, the cells become dependent on GDNF, and RUNX1 expression is maintained [5]. Our experimental design is based on the above studies in rodents.

In summary, we show that hEPI-NCSC can be differentiated into capsaicin-sensitive neurons that express SP and CGRP as well as other pertinent markers. The neurons can be generated in large numbers and with high efficiency, i.e. with antibody marker expression in virtually all cells within a short period of time (18 days) and without the need for cell purification or genetic manipulation. These peptidergic nociceptive neurons promise to be attractive candidates for the study of capsaicin-mediated pain transduction and use in drug discovery and disease modelling.

## Materials and methods

### Ethical approvals

All experiments with human skin were performed in accordance with relevant guidelines and regulations and with the approval of the Northumberland Research Ethics Committee of the NHS Health Research Authority and the Faculty of Medical Sciences Ethics Committee of Newcastle University (REC REF: 08/H0907/1). De-identified biopsies of full-thickness pubic hairy skin from second Caesarean sections were obtained with written informed consent from all subjects. Human foetal tissue at 12 post conception weeks (PCW) was obtained from the Joint MRC/Wellcome Trust (grant # 099175/Z/12/Z) Human Developmental Biology Resource (HDBR) (http://www.hdbr.org) with appropriate maternal written consent and approval from the Newcastle and North Tyneside NHS Health Authority Joint Ethics Committee. HDBR is regulated by the UK Human Tissue Authority (HTA; www.hta.gov.uk) and operates in accordance with the relevant HTA Codes of Practice. The Illumina gene expression profile was deposited at NCBI Gene Expression Omnibus

### Culture media

The composition of expansion (XP) medium is described in Clewes et al (2011) [27] and sources are provided in S1 Table. Pre-differentiation medium consisted of Neurocult NS-A medium, NS-A *Proliferation* Supplement, fibroblast growth factor (rhFGF2; 10 ng/ml), epidermal growth factor (rhEGF; 20 ng/ml), stem cell factor (rhSCF; 5 ng/ml), brain derived neurotrophic factor (rhBDNF; 20 ng/ml), neurotrophin-3 (rhNT-3; 10 ng/ml), heparin (2 μg/ml), SITE+3, β-mercaptoethanol (10 μM), foetal bovine serum (FBS; 1%), and GlutaMAX. Differentiation medium consisted of NeuroCult NS-A base medium, NeuroCult NS-A *Differentiation* Supplement, nerve growth factor (rh β NGF; 20 ng/ml), db cAMP (1 mM), B27 Supplement without retinoic acid (1x), β-mercaptoethanol (10 μM), ascorbic acid (200 μM), FBS (1%), GlutaMAX (1x), penicillin/streptomycin (1x). Hepatocyte growth factor (rhHGF) was used at 50 ng/ml. Detailed source information is provided in S1 Table.

### hEPI-NCSC cultures

All experiments were performed in accordance with relevant guidelines and regulations and with the approval of the Northumberland Research Ethics Committee of the NHS Health Research Authority and the Faculty of Medical Sciences Ethics Committee of Newcastle University (REC REF: 08/H0907/1). De-identified hairy skin biopsies were obtained with written informed consent from all subjects. hEPI-NCSC were isolated from human hair follicles exactly as we have described previously [27]. Briefly, anagen hair follicles were dissected and the bulge section placed into adherent culture. hEPI-NCSC emigrated from bulge explants within 6–10 days. Bulge explants were removed manually, leaving the hEPI-NCSC in adherent culture. They were subcultured three days after onset of emigration, expanded for six days exactly as we have described and cryopreserved [27]. Cryopreserved cells were thawed, cultured in XP medium for several days, and subcultured onto Matrigel using various base media depending on the stage of differentiation that were supplemented with small molecules, pertinent growth factors and other reagents as described above, below in the experimental design section, and in S1 Table.

### Indirect immunocytochemistry

Indirect immunocytochemistry was performed exactly as we have described previously [27, 28, 29]. Briefly, cultures were fixed with 4% paraformaldehyde, rinsed, blocked, and incubated overnight in the cold with primary antibodies in 0.1% Triton X100. Subsequently, cultures were rinsed, incubated with secondary antibodies, rinsed again and mounted with ‘Vectashield hard set mounting medium with DAPI’. Fluorescence was observed with an Zeiss Axio Imager Z1 fluorescence microscope. Secondary antibodies were designed for multiple labelling with minimal cross-reactivity with human, bovine, horse, rabbit/mouse, and swine serum proteins. Negative controls did not show any immunofluorescence. The list of reagents and sources is provided in S1 Table.

### RNA isolation and real-time polymerase chain reaction (qPCR)

Total RNA was extracted from snap-frozen homogenized undifferentiated hEPI-NCSC and from D8 differentiating cells. RNA purity determined using an Agilent 2100 Bioanalyser (hEPI-NCSC, RIN 8.9; neurons, RIN 9.4). For qPCR, 900 ng total RNA was incubated with DNaseI and processed exactly as we have described previously [27, 28, 29]. Ct values for targets were normalized to the average Ct value of GAPDH. Fold changes in expression were calculated using the ΔΔCt method. Statistical analysis was performed using the Student’s t-test. The list of reagents and sources is provided in S1 Table.

### Calcium imaging

Cells were loaded with Fluo-4 AM (2μM, Life Technologies) at 37°C, 5% CO_2_ in culture media supplemented with 0.02% pluronic F-127 (Life Technologies) for 25 minutes. The medium was then removed and replaced with imaging buffer (10 mM HEPES, 5.9mM potassium chloride, 135 mM sodium chloride, 11.5 mM glucose, sodium hydroxide (pH 7.3), 1.2 mM magnesium chloride, 1.5mM calcium chloride). Prior to imaging, cells were left to equilibrate at 37°C for 20 minutes. Imaging was performed with a Nikon A1R confocal microscope with the stage kept at 37°C throughout. Imaging was performed at 20x magnification (Nikon Plan Apo 0.75 NA, VC) at 488 nm and in resonant scanning mode (bi-directional, at 15 frames per second with double line averaging).

Cells were observed initially for 1–2 minutes to estimate dye photo-bleaching and to record any spontaneous activity. Calcium fluxes were induced using capsaicin (100nM or 1 μM) to activate the TRPV1 receptor or by high potassium challenge (10mM HEPES, 85.9mM potassium chloride, 55mM sodium chloride, 11.5 mM glucose, 1.2 mM magnesium chloride, 1.5 mM calcium chloride and sodium hydroxide to pH 7.3). The calcium ionophore ionomycin (10μM) was used at the end of the experiments as a control for dye loading [30]. All experiments were performed with cultures between D18 and D22.

### Image analysis

Images were analysed using ‘FIJI’ (FIJI Is Just ImageJ) software [31]. Each file was opened as an image stack. The background was corrected for by subtracting the mean intensity of an area with no activity from all the images in the stack. A composite image of all images in a stack showing the maximal intensity of each pixel was then generated using the ‘Z project’ feature. To generate a map of the cells and areas of activity in processes, the ‘threshold’ feature was applied to the composite image, which allowed for the ‘analyse particles’ feature to be used; it selects regions of interest (ROIs) above a size specified by the user, to draw ROIs around all cell bodies and areas of activity in neuronal processes. The original image stack was then divided into sub-stacks for each solution added. The ROIs were subsequently mapped onto the original image stack and the multi-measure tool was used to measure the intensity of the ROIs across the whole image stack. This created a list of numbers that corresponded to the mean intensity of each ROI at a particular frame, which was transferred to Microsoft Excel. These values were then averaged to give the mean intensity of all the ROIs measured at a given frame. Intensity is used as a marker for fluorescence. A value for the baseline fluorescence (F0) was derived by averaging the intensity of the first 10 frames of the stack. The time at which each frame was taken was calculated using the frame rate and the times at which each solution was added. The mean intensity of individual ROIs measured at a given time point (F) was divided by F0 and plotted on a line graph to show changes in fluorescence in relation to F0 over time. Screenshots from FIJI during points of activity were taken between a specified number of frames to visualise changes in fluorescence intensity over time.

### Human foetal dorsal root ganglia isolation and immunohistochemistry

Cross-sections containing 2–3 thoracic segments were prepared from tissue at 12 PCW, the spinal cord with attached DRGs dissected, fixed with 4% paraformaldehyde for 60 minutes and rinsed with phosphate buffered saline. Tissue was placed into cryomolds with OCT compound, quick-frozen on dry ice and cryosectioned (10 μm). Sections were treated with 0.4% Triton X-100 for 20 minutes at room temperature, rinsed exhaustively and blocked with 5% normal goat serum for 1 hour. The remaining steps of immunohistochemistry were performed as described above. Details on reagents are provided in S1 Table.

## Results

### Modulate signalling pathways to differentiate hEPI-NCSC into peptidergic nociceptive neurons

hEPI-NCSC are neural crest derived somatic stem cells that are located in the ‘bulge’ of hair follicles. The bulge is a stem cells niche in which epidermal stem cells reside, which give rise to skin, hair and sebaceous glands. Intermixed with epidermal stem cells are neural crest derived multipotent stem cells, as we have described in the mouse [32], in the dog [33] and with human tissue [27]. The neural crest origin was first determined in the WNT1-cre/R26R mouse in which all neural crest derived cells are indelibly marked by their expression of beta-galactosidase [32] and by LongSAGE gene expression profiling [34]. The neural crest origin of canine and human EPI-NCSC was determined by gene expression profiling and quantitative polymerase gene reaction (qPCR) [27, 33]. The stemness and multipotentiality was determined by in vitro clonal analyses in all three species. hEPI-NCSC were isolated by dissection of anagen hair follicles in the dermal part of full-thickness hairy skin biopsies. For isolation and self-purification of hEPI-NCSC, advantage was taken of the migratory ability of neural crest cells. In adherent culture, hEPI-NCSC emigrated from bulge explants within a few days, and concomitantly they proliferated rapidly. The cells were detached and subcultured in adherent culture for ex vivo expansion into millions of stem cells and cryopreserved for later use, exactly as described previously [27]. The cells continued to proliferate during the first six days (D -3 to D +2) of the differentiation protocol described in this study.

**Fig 1:**
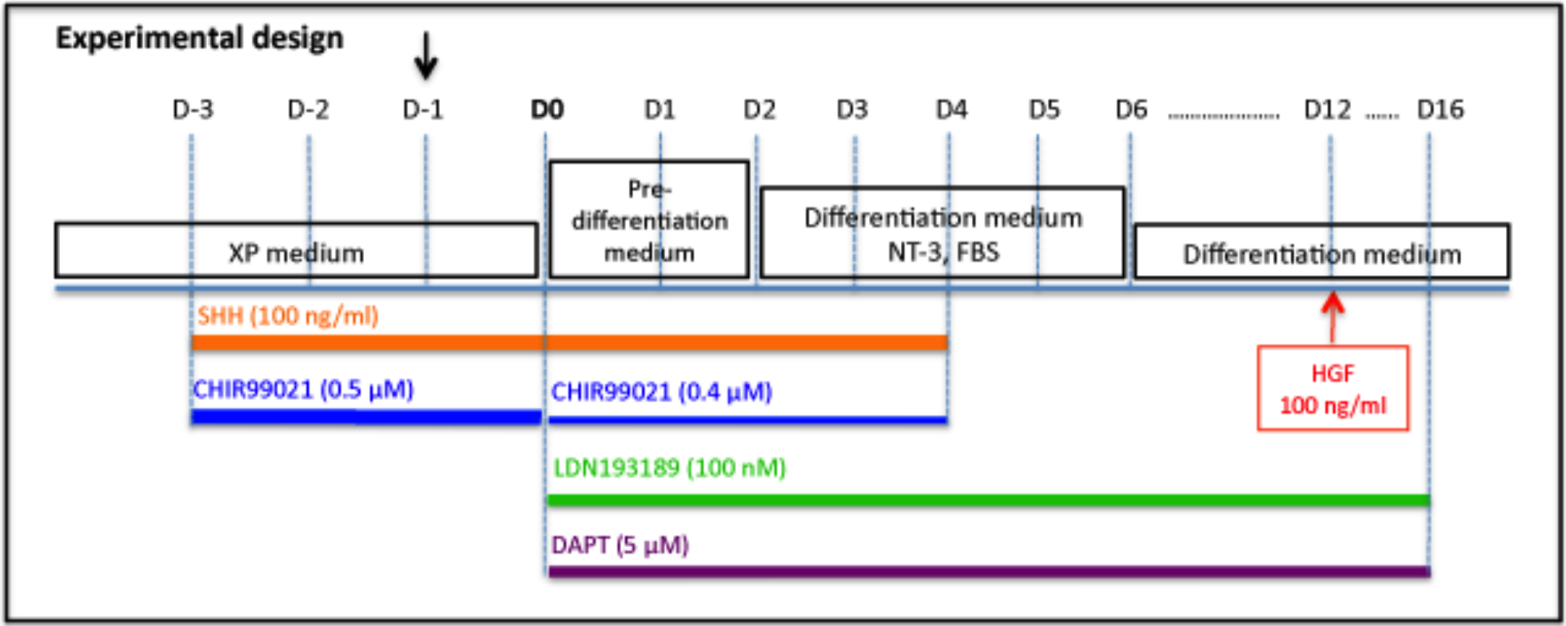
Experimental design. Cells were cultured for several days in expansion (XP) medium. At day minus 3 (D-3) sonic hedgehog (SHH) and CHIR99021 were added to the culture medium. Cells were subcultured into XP medium that contained SHH and CHIR99021 (0.5 μM) at D-1. At D0 the culture medium was changed to Pre-differentiation medium and was supplemented with CHIR99021 (0.4 μM), LDN193189 (100 nM) and DAPT (5 μM). At D+2, the culture medium was changed to Differentiation medium that was supplemented with NT-3 and 1% FBS. SHH and the three small molecules were also added. At D+4, SHH and CHIR99021 were removed. At D+6, the culture medium was changed to Differentiation medium, that is in the absence of NT-3 and FBS. HGF (100 ng/ml) was added on D+12. The culture medium was exchanged every 48 hours.

Here we describe the experimental procedure to generate peptidergic nociceptive sensory neurons from hEPI-NCSC. The term ‘neuron’ is used here based on the fact that at the end of the differentiation period, the cells were post-mitotic and had neuronal morphology. The experimental design was based on the pertinent literature from studies in rodents and on pilot experiments (not shown). Here we present the experimental design that lead to robust data (Fig 1). We found that exposure of the cells to SHH and enhancing the canonical WNT signalling pathway was essential. Accordingly, at differentiation day minus three (D -3), SHH (100 ng/ml) and the GSK-3 inhibitor CHIR99021 (0.5 μM) were added to the XP medium. Starting on D 0, cells were cultured in Pre-differentiation Medium in the continued presence of SHH and CHIR99021. The CHIR99021 concentration was lowered in order to keep DMSO concentration at 0.1%. In pilot studies we also found it important to inhibit BMP signalling in order to guide the cells away from the sympathetic neuron cell lineage. Additionally, it was essential to block Notch/Delta signalling, which inhibits NGN1/2 expression. Therefore, at D0 also, the ALK inhibitor LDN193189 (100nM) and the γ-secretase inhibitor DAPT (5μM) were first added. At D+2, the culture medium was changed to Differentiation Medium with the further addition of NT-3 for four days to maintain TRKA and BRN3a expression and 1% FBS. FBS (1%) was added again once for 24 hours on D +16. rhHGF (100 ng/ml) was first added on D+12 and exchanged every 48 hours until the end of the culture period on D+21, at which point calcium imaging was performed. Using an alternative protocol that did not contain SHH and CHIR99021 did not yield high intensity cytoplasmic or nuclear NGN2 and RUNX1 immunoreactivity (data not shown). However, the SHH treatment could be shortened from seven to two days, and CHIR99021 treatment from seven to four days. Similarly, LDN193189 seemed no longer essential after two days. Overall, we found that the experimental design shown in Fig 1 is reliable in leading to robust differentiation of hEPI-NCSC into capsaicin-sensitive peptidergic neurons.

### Transition from stem cell morphology to bipolar sensory neuron morphology during in vitro differentiation

**Fig 2:**
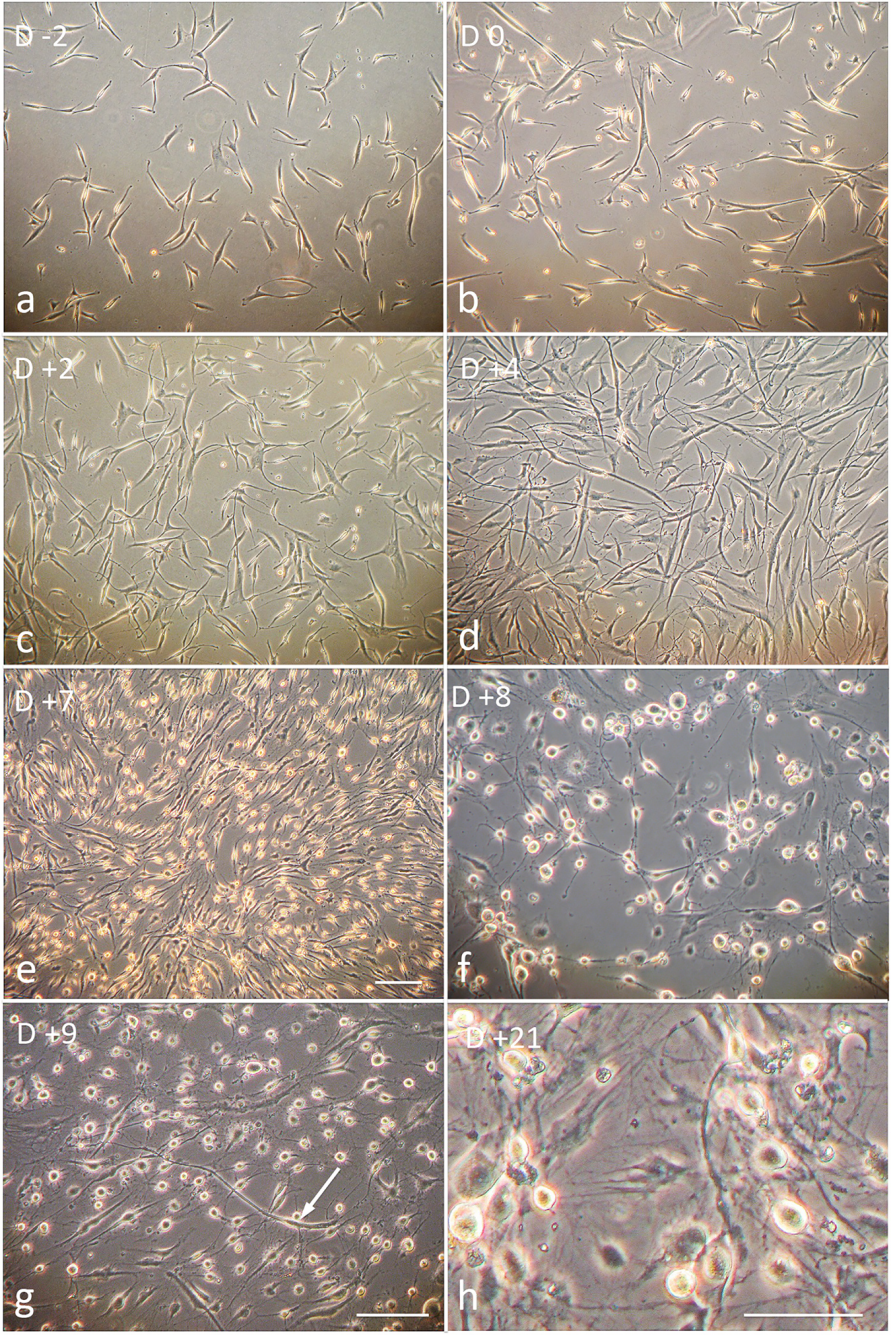
Cell morphological changes during in vitro differentiation. (a) Cells during ex vivo expansion had the stellate morphology typical for neural crest cells. (b-c) SHH and CHIR99021 treatment had no noticeable influence on cell morphology. (a-d), During the early phases of the protocol, rounded cells represent dividing cells. (d) By D +4, cells had started to elaborate short processes. (e) Between D +6 and D +7, most cell bodies started to round up. (f) Most cells had the morphology of bipolar neurons. (g) By D +9, most remaining elongated cells started to acquire a round cell body (arrow). (e–h) In contrast to earlier phases in the protocol, rounded cells represent neuronal somata during the later phases of differentiation. Due to the morphological change from flattened cells to rounded somata, more space appeared between cells. There was no significant cell death. (h) By D +21, some neurons were lost because the round cell bodies easily detached from the substratum and the long neurites, which were never attached to the substratum, tended to break during culture medium exchanges. Coating the cells with a 3D collagen matrix might solve this technical problem in future experiments. However, many cells, both bipolar neurons and flattened cells, persisted throughout the three week-long culture period. We did not pursue cultures beyond three weeks even though that could be done, as the phenoptype was stable. “D”, day in culture according to protocol shown in Fig 1. Bars, (a–g), 50 μm; (h), 50 μm.

One important aspect of sensory neuron differentiation is the transition from stem cell morphology to bipolar, and ultimately pseudo-unipolar, neurons. We monitored this morphological change during in vitro differentiation with phase contrast imaging. During the ex vivo expansion period, cells had the stellate morphology typical for neural crest cells (Fig 2A), which as expected did not noticeably change with SHH and CHIR99021 treatment (Fig 2B and 2C). By Day 4 (D+4) of differentiation, however, cells had started to extend short processes (Fig 2D) and by D+7/8 greater than 90% of cells had the rounded soma and bipolar characteristics of immature sensory neurons (Fig 2E and 2F). Most of the remaining approximately 10% of elongated cells started to acquire sensory neuron morphology by D9 (Fig 2E, 2F and 2G). At around two weeks of differentiation, many rounded somata started to detach from the Matrigel substrate. These neurons died over time due to rupture sustained by the neurites during medium exchanges. Cells became smaller with progressing differentiation. A single 24-hour treatment with 1% FBS (D+16) restored cell size and also caused flattening of many cells. The flattened morphology did not affect antibody marker expression or capsaicin sensitivity. Many cells with sensory neuron morphology survived for three weeks or longer (Fig 2H). In summary, the data indicate that with progressing in vitro differentiation hEPI-NCSC assumed the bipolar morphology of immature primary sensory neurons.

### Time-course of peptidergic nociceptive sensory neuron marker expression during in vitro differentiation

**Fig 3:**
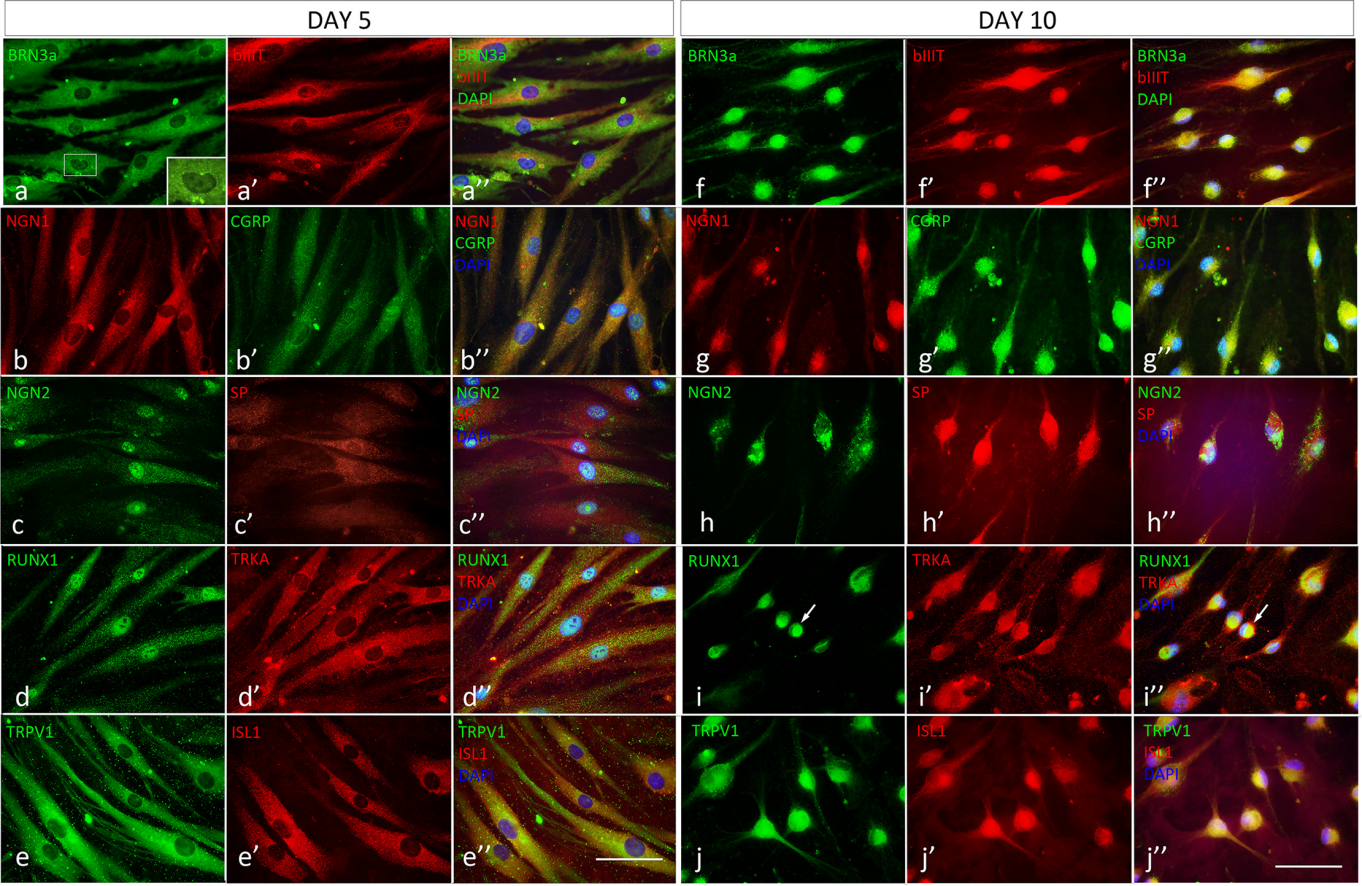
Marker expression during in vitro differentiation. ***Day 5 of differentiation*** (a–a” to e–e”). (a–a”) On D5 all cells were immunoreactive for BRN3a (green) in the nucleus (inset) and the cytoplasm, and all cells were positive for β-III tubulin (red). (b–b”) NGN1 was expressed in the cytoplasm of all cells (red) and there was faint CGRP immunofluorescence (green) in all cells. (c–c”) NGN2 was expressed predominantly in the nucleus in all cells (green) and there was distinct punctate SP immunoreactivity (red) in most cells. (d–d”) All cells expressed distinct nuclear RUNX1 immunoreactivity (green) and they were TRKA immunoreactive (red). (e–e”) There was intense TRPV1 immunoreactivity (green) in all cells and they also expressed ISL1 (red). (a”, b”, c”, d”, e”, merged images) ***Day 10 of differentiation*** (f–j”). By D10, cells had assumed bipolar neuron morphology and most cells had an eccentric nucleus typical for sensory neurons. (f–f”) All cells expressed BRN3a and β-III tubulin. (g–g”) NGN1 was expressed in all cells but with lower fluorescence intensity than on D5 (red) whereas CGRP immunofluorescence had become more intense (green). (h–h”) NGN2 was still expressed but was predominantly localized in the cytoplasm (green), whereas SP immunoreactivity was intense (red). (i–i”) RUNX1 immunoreactivity was present, but was localized in the cytoplasm (e.g. arrow) in most cells (green); all cells still expressed TRKA. (j–j”) All cells were intensely TRPV1 immunoreactive (green) and also expressed ISL1 (red). (f”, g”, h”, I”, j”, merged images. Bars, (a–e”) 50 μm; (f–g”) 50 μm.

**Fig 4:**
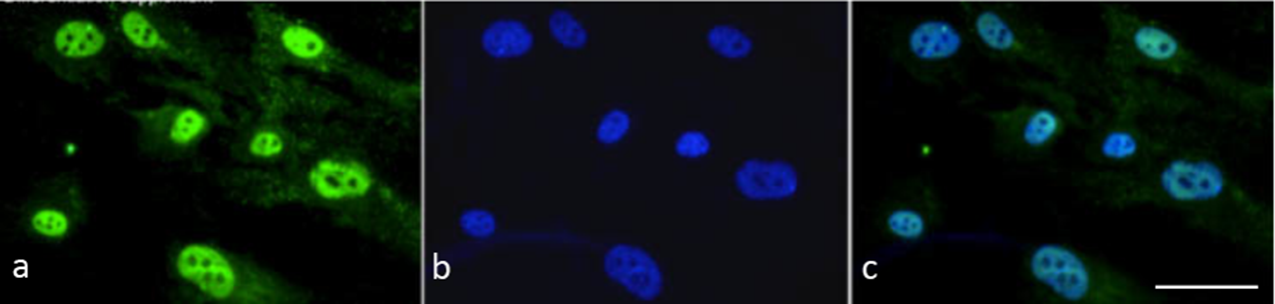
BRN3a nuclear localisation. (a) BRN3a immunoreactivity (green fluorescence) was predominantly nuclear in the continued presence of NT-3, BDNF and FBS (1%). (b) DAPI nuclear stain. (c) Merged images. Bar, 50 μm.

We monitored progression of cell differentiation by immunocytochemistry, using a panel of 10 antibodies that recognise pertinent peptidergic nociceptive sensory neuron markers. BRN3a was expressed in all cells by D5 of differentiation in the nuclei and in the cytoplasm and it remained expressed throughout the culture period (Fig 3A and 3A”; 3F and 3F”). Likewise, all cells expressed β-III tubulin throughout the culture period (Fig 3A’ and 3A”; 3F’ and 3F”). There was strong cytoplasmic Neurogenin1 (NGN1) immunofluorescence throughout the culture period (Fig 3B and 3B”; 3G and 3G”). Weak CGRP fluorescence was first observed by differentiation day D5 (Fig 3B’ and 3B”). CGRP immunofluorescence increased in intensity with progressing differentiation and was intense by D10 (Fig 3G’ and 3G”). Notably, there was strong nuclear Neurogenin 2 (NGN2) immunofluorescence by D5 (Fig 3C and 3C”), which as expected declined by D10 (Fig 3H and 3H”). Weak Substance P (SP) immunofluorescence was detectable already by D5 (Fig 3C and 3C”) and increased in intensity over time (Fig 3H’ and 3H”). There was intense nuclear RUNX1 immunofluorescence by D5 (Fig 3D and 3D”), which in some cells became cytoplasmic by D10 (Fig 3I and 3I”) and progressively decreased in intensity in the presence of hepatocyte growth factor (HGF) for nine days (data not shown). There was strong TRKA Immunofluorescence by D5 (Fig 3C’ and 3C”), which was maintained throughout the culture period (Fig 3I’and 3I”). There was strong TRPV1 immunofluorescence already by culture D5 (Fig 2E and 2E”) and cells were also positive for Islet-1 (ISL1) (Fig 3E’ and 2E”). Both, TRPV1 and ISL1 immunofluorescence persisted throughout the culture period (Fig 3J–3J”). An increase in expression of the TRPV1, SP and CGRP genes was also observed at the RNA level by qPCR (Table 1). TRPV1 gene expression was increased in neurons 3.2-fold, SP 2.4-fold and CGRP 2.5-fold compared to hEPI-NCSC (Table 1). While in addition to nuclear expression there was also cytoplasmic immunofluorescence of BRN3a with the current experimental design, BRN3a was predominantly expressed in the nucleus in the continued presence of NT-3, BDNF and FBS (Fig 4).

**Table 1:** Quantitative polymerase chain reaction.

Gene	Fold-increase in neurons compared to hEPI-NCSC±S.E.
TRPV1	3.2±0.5 (p = 0.03)
Substance P	2.4±0.3 (p = 0.04)
CGRP	2.5±0.5 (p = 0.08)

***Note***: n = 3.

Since immunocytochemistry is not amenable to precise quantification, we characterised the stem cells by comparing levels of expression of stem cell genes and neural crest cell genes to genes known to be expressed in nociceptive peptidergic neurons, using data from our Illumina gene expression profile (NCBI Gene Expression Omnibus <http://www.ncbi.nlm.nih.gov/geo/>, GEO accession number GSE61273). The stem cell genes and neural crest cell genes NES, SNAI1, MSI1, TWIST1 and NOTCH1 were highly likely expressed with detection p-values between 0.00 to 0.08. In contrast, the sensory neuron genes NGN1 (NEUROG1), NGN2, RUNX1, BRN3a (POU4F1), CGRP (CALCA), SP (TAC1) and TRPV1 were highly unlikely to be expressed in the stem cells with detection p-values between 0.46 to 0.90 (Table 2). These data show that hEPI-NCSC do not express any sensory neuron genes at detectable levels.

**Table 2:** Expression levels of stem cell/neural crest cell and neuronal markers in hEPI-NCSC.

Gene	Detection p-value
*Neural crest stem cell and general stem cell markers in hEPI-NCSC*	
NES	0.00
SNAI2	0.00
MSI1 (Musashi)	0.08
TWIST1	0.00
NOTCH1	0.00
*Nociceptive peptidergic sensory neuron markers in hEPI-NCSC*	
NGN1 (NEUROG1)	0.46
NGN2	0.73
RUNX1	0.88
BRN3a (POU4F1)	0.73
CGRP (CALCA)	0.90
SP (TAC1)	0.82
TRPV1	0.81

***Note*:** Data were obtained by Illumina gene expression profiling. A detection p-value of zero indicates the highest possible probability with which a gene is expressed. A detection p-value of 1 indicates the lowest possible probability with which a gene is expressed.

### Capsaicin-sensitivity in differentiated sensory neurons

**Fig 5:**
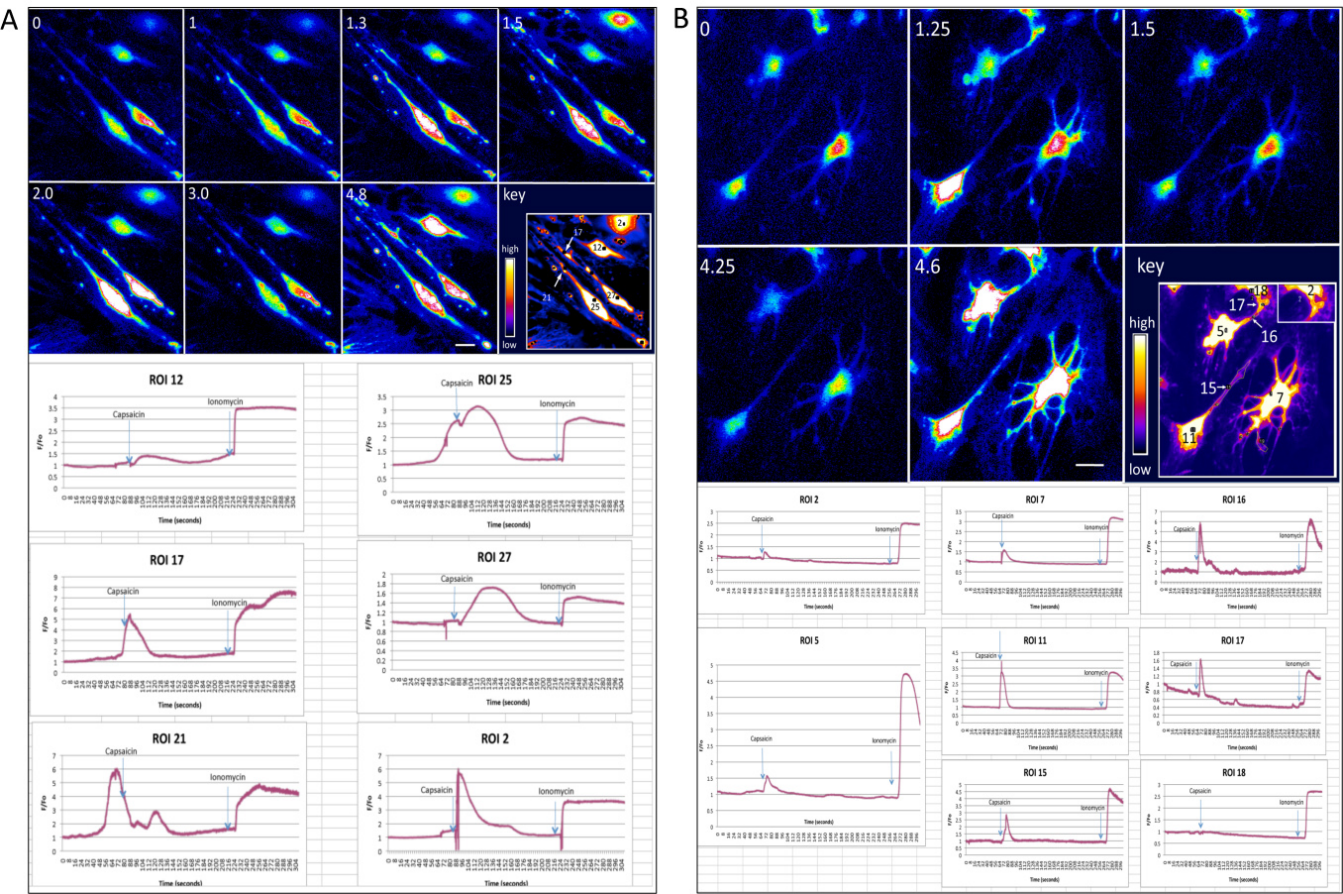
Response to capsaicin. **Panel A**, Time course of Fluo-4 intensities before and after addition of Fluo-4. Upper panel, four cell bodies are visible (D21 of culture). Numbers in upper left of individual images correspond to time in minutes of experiment. (0; minute zero) Images captured before addition of capsaicin. (min.1) Before the addition of capsaicin, spontaneous activity was observed in the two cells at the lower left (region of interest 25 and 27). Capsaicin was added at min 1.25. At 1.3 min and 1.5 min, intensity of fluorescence increased in the two cells at the lower left and the cell at the upper right reached its peak of reacting to capsaicin. (min. 2.0) By minute 2.0, the two neurons at the lower left reached their peak of fluorescence, as did the cell above. (3.0) By 3 minutes, intensity of fluorescence had decreased in all cells. Ionomycin was added at minute 3.4.; image was captured at min. 4.8. All four cells responded to ionomycin with calcium flux. Lower panel, F/F0 plots of various ROIs, as identified in the image marked ‘key’. ROI 21 showed spontaneous activity prior to the addition of capsaicin, which was closely followed by activity in ROI 25 of the same cell, whereas ROI 21 responded again with a delay. All cells and ROIs responded to ionomycin. **Panel B**, Capsaicin sensitivity of D21 cells in a different experiment and culture. Capsaicin was added at minute 1.1. Seven of 8 ROIs showed a fast response to capsaicin. All showed a robust response to ionomycin. Cells had a flattened appearance due to the 24-hour exposure to FBS. Morphology did not affect capsaicin-responsiveness. Bars, Panels A and B, 25 μm.

We tested capsaicin-sensitivity in D+21 in vitro generated nociceptive peptidergic neurons by calcium imaging using Fluo-4 AM (Fig 5); 29.8±11.6% of cells responded to 100 nM capsaicin and 29.9±11.5% of cells responded to 1 μM capsaicin (Table 3). An average of 38.0±14.0% of cells responded to high concentrations of KCl (Table 3). As a control for dye-loading, cells were exposed to the calcium ionophore ionomycin at the end of the experiment; 86.6±3.61% of cells responded (Table 3). Fig 5, Panel A shows capsaicin responses as time-course of Fluo-4 AM intensities. Cells are shown in the upper panel. Numbers at the upper left of each image represent time in minutes. Capsaicin (1 μM) was added at minute 1.25. The regions of interest (ROI) are defined in the image termed “key”. Four cell bodies are visible, defined as ROI 25, 27, 12, and 2. Spontaneous activity was observed in the processes of the neurons at the lower left (cell bodies ROI 25 and 27). All four neurons responded to capsaicin, albeit to different degrees, as illustrated by a transient increase in fluorescence intensity. All four cells responded to ionomycin at the end of the experiment (Fig 5A; 4.8 minutes). In the lower panel, the F/F0 plots for the six ROIs over the time-course of the experiment are shown. Blue arrows indicate time of addition of capsaicin and ionomycin. All cell bodies (ROI 25, 27, 2), except ROI 12, responded to capsaicin but showed different amplitudes, with the highest and fastest response observed in ROI 2. An equally high response was seen in the process ROI 17. By contrast, neurons in which spontaneous activity was observed prior to the addition of capsaicin responded with a delay. Minute 4.8 shows fluorescence intensity after addition of ionomycin. In Fig 5, Panel B, a time-course of calcium flux in response to capsaicin in D+21 cells from a different experiment is shown. In the upper panel fluorescence in cells is shown. The lower panel shows the F/F0 plots corresponding to the marked ROIs. Capsaicin was added at minute 1.1. All ROIs, except ROI 18, show a fast response. All ROIs show a robust response to ionomycin at the end of the experiment (minute 4.6). Overall, 12/21 ROIs examined showed an immediate response to capsaicin. Further electrophysiological experimentation needs to be conducted to characterize the neurons’ activity in detail.

**Table 3:** Calcium imaging.

Solution added	Responding cells Mean of % ± SEM	Maximal F/F0 Mean ± SEM
None	7.8±4.1* (p = 0.033)	1.31±0.17* (p = 0.07)
Capsaicin (100 nM)	29.8±11.6 (p = 0.99)	1.10±0.01* (p = 0.01)
Capsaicin (1 μM)	29.9±11.5	1.91±0.27
Potassium Chloride (80 mM)	38.0±14.0 (p = 0.66)	1.77±0.27 (p = 0.61)
Ionomycin (10 μM)	86.6±9.6* (p = 0.002)	3.61±0.20* (p<0.001)

*Note*: n = 67–134.

*) P-values statistically different from 1 μM Capsaicin values.

### Brn3a, ISL1. TRPV1, Nestin, neurofilament and TRKA expression in human foetal dorsal root ganglia (DRGs) at 12 weeks post conception (WPC)

**Fig 6:**
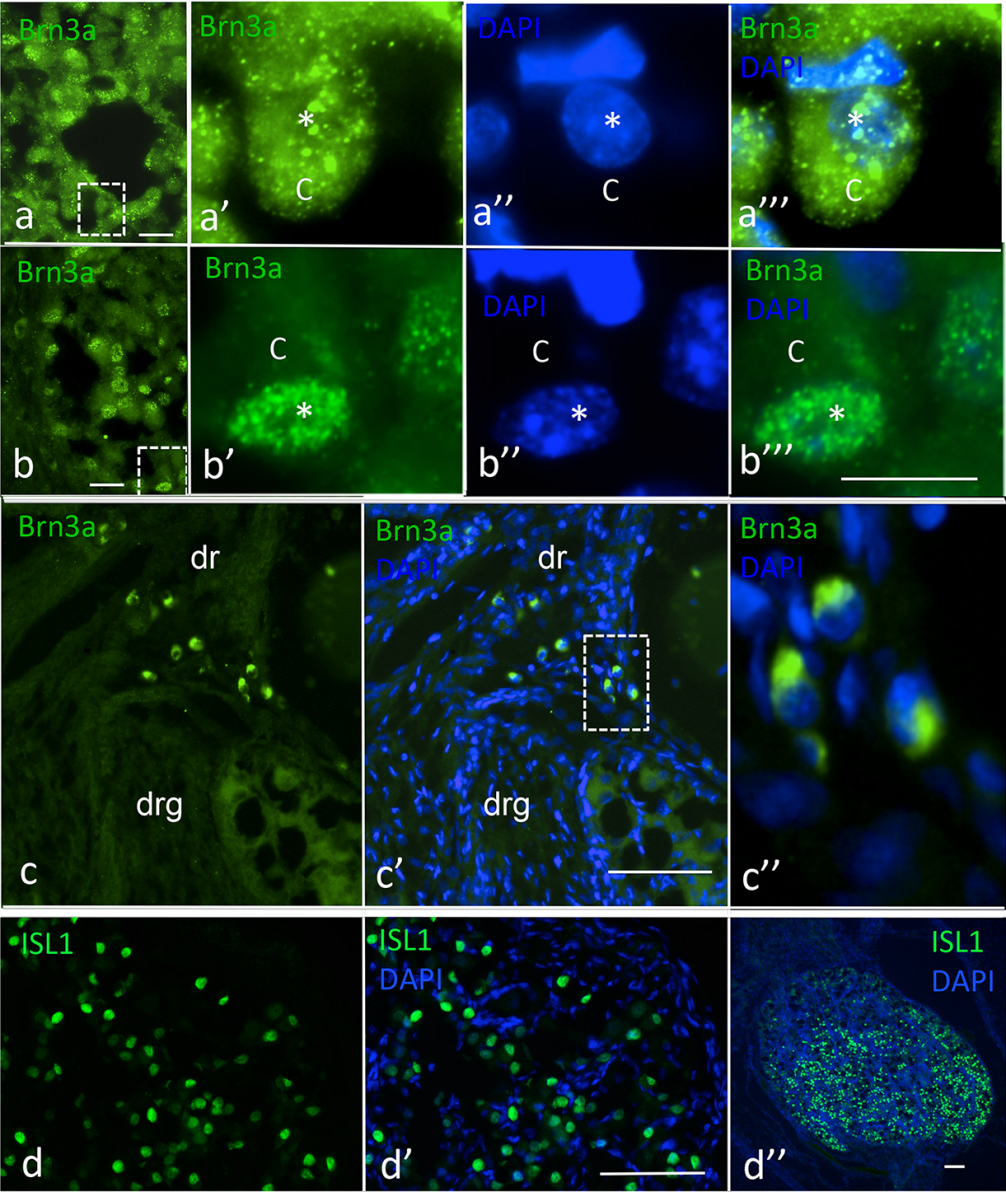
BRN3a and ISL1 immunofluorescence in thoracic human foetal DRGs at 12 PCW. (a–a”‘) Cytoplasmic Brn3a immunofluorescence in 12 PCW DRG. (a) Overview of ganglionic area. (a’) Larger magnification of boxed area in (a); asterisk, location of nucleus; c, cytoplasm. (a”) DAPI nuclear stain of same area as in (a’). (a”‘) Merged images. (b–b”‘) Nuclear expression of Brn3a in 12 PCW DRG. (b) Overview of ganglionic area. (b’) Nuclear expression of Brn3a; asterisk, nucleus; c, cytoplasm. (b”) DAPI nuclear stain. (b”‘) Merged images. (c-c”) Cytoplasmic expression of Brn3a in boundary cap cells; (c) Brn3a expression; dr, dorsal root; drg, dorsal root ganglion. (c’) Brn3a and DAPI merged images. (c”) Larger magnification of boxed area in (c’). (d–d”) ISL1 expression in 12 PCW DRG. (d) Green ISL1 immunofluorescence. (d’) ISL1 and DAPI merged images of same area shown in (d). (d”) ISL1 (green) and DAPI (blue) merged images of entire section of the same ganglion. Bars; (a), (b), 50 μm; a’ a”‘ and b’–b”‘, 25 μm; (c–c’), 50 μm; (d–d’), 50 μm.

**Fig 7:**
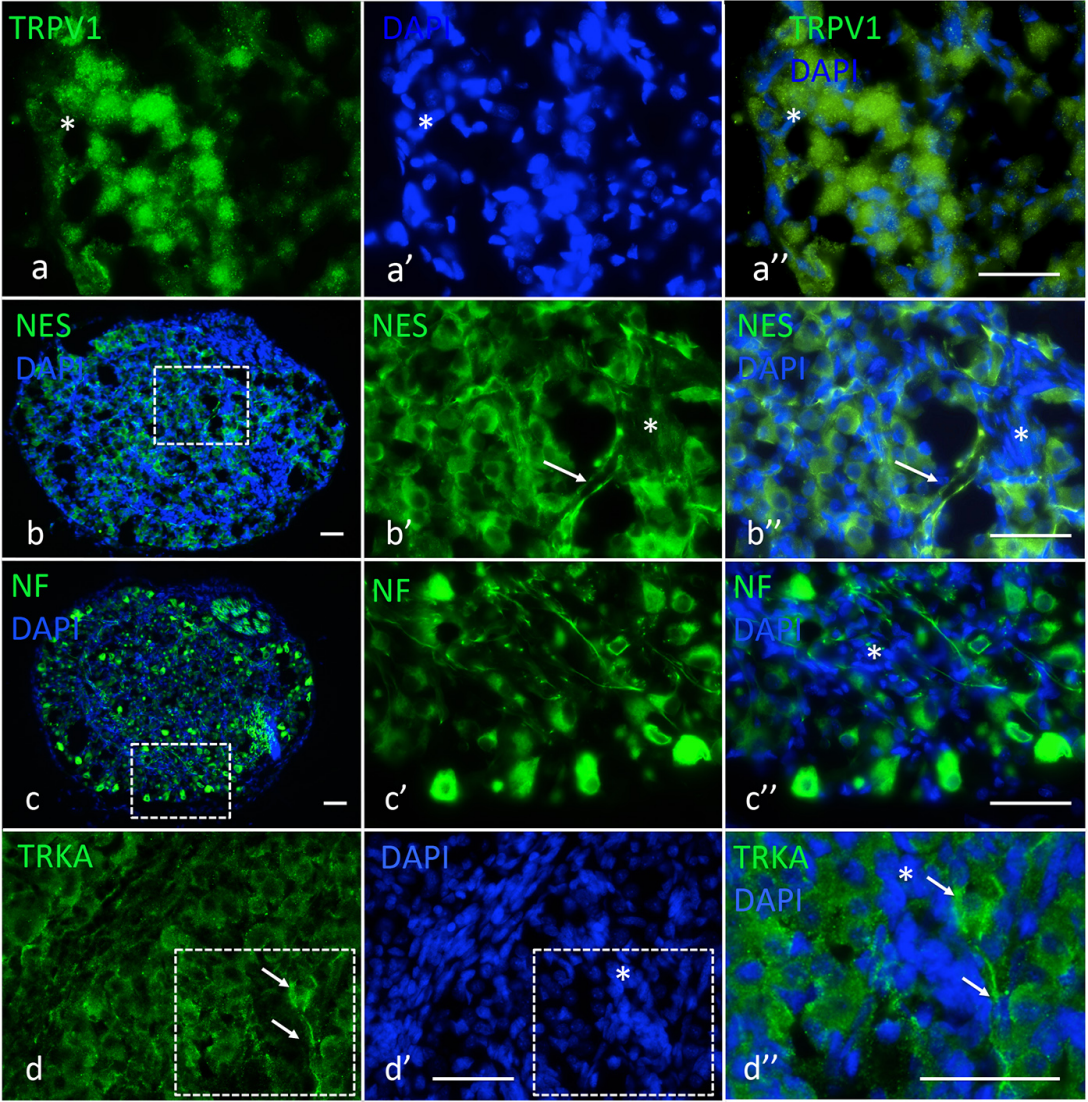
TRPV1, nestin (NES), neurofilament (NF) and TRKA expression in 12 PCW thoracic human DRG. (a–a”) TRPV1 immunofluorescence. (a) TRPV1; (b) DAPI nuclear stain of same area. (a”) TRPV1 and DAPI merged images of same area with TRPV1 Immunofluorescence in neurons (round nuclei); asterisk, an example of a non-neuronal TRPV1-negative cell. (b–b”) Nestin expression. (b) NES and DAPI merged images of section through ganglion. (b’) NES immunofluorescence of boxed area in (b); arrow, example of NES-positive neurites; asterisks area of NES negative non-neuronal cells with slender, elongated nuclei. (c–c”) Neurofilament immunoreactivity. (c) Overview of section of ganglion, NF and DAPI merged images. (c’) NF immunofluorescence in neurons; higher magnification of boxed area in (c). (c”) NF and DAPI merged images of same area as in (c’); asterisk, area of non-neuronal cells, which are NF-negative. (d–d”) TRKA immune fluorescence. (d) TRKA immunoreactivity; most neurons appear to express TRKA; arrows, example of a neuron with neurite. (d’) DAPI nuclear stain of same area as in (d); asterisk, area with non-neuronal cells. (d”) Merged images of boxed area in (d) and (d’) at higher magnification; arrows, TRKA-positive neuron with neurite; asterisk, non-neuronal cells, which are TRKA-negative. Bars, (a–a”), 50 μm; (b), 50 μm; (b’–b”), 50 μm; (c), 50 μm; (c’–c”), 50 μm; (d–d’), 50 μm; (d”), 50 μm.

In order to correlate the in vitro data with equivalent neurons in vivo and because little is known about marker expression in human foetal DRG development, we performed a comparative study on marker expression in foetal human primary sensory neurons in DRGs at 12 WPC by indirect immunocytochemistry. In thoracic DRGs, BRN3a immunoreactivity was present at different intensities in different cells (Fig 6). Some cells showed cytoplasmic BRN3a immunofluorescence (Fig 6A–6A”‘), whereas in other areas of the ganglia, BRN3a immunofluorescence had nuclear localization (Fig 6B–6B”‘). Figs (a) and (b) show an overview over a ganglionic section, whereas a’–a”‘ and b’–b”‘ show the boxed area in (a) and (b) at higher magnification. Furthermore BRN3a immuno-positive boundary cap cells, with cytoplasmic BRN3a expression, were present at the boundary between the DRG and the dorsal root (Fig 5C–5C”). As expected, ISL1 immunoreactivity was present in many but not all DRG neurons (Fig 6D–6D”). TRPV1 immunoreactivity was observed in some neurons but not in others and not in non-neuronal cells (Fig 7A–7A”). As expected, because glia cells differentiate before neurons, neurons still expressed nestin immunofluorescence (NES; Fig 7B–7B”, e.g. arrow), whereas non-neuronal cells were NES-negative (Fig 7B–7B”, asterisk). Neurofilament (NF) immunoreactivity was present in neurons but not in non-neurons as expected (Fig 7C–7C”, e.g. asterisk). TRKA immunoreactivity was widespread in neurons, more intense in some neurons, and absent in non-neuron cells (Fig 7D–7D”). Fig 7D” shows the boxed area in (d) and (d’) at higher magnification. A TRKA-positive neuron with process (arrows) and TRKA-negative non-neuronal cells (e.g., asterisk) are visible.

## Discussion

The overall result of this study is that capsaicin-sensitive peptidergic nociceptive primary sensory neurons that express TRPV1, SP, CGRP, and other pertinent markers, can differentiate efficiently from hEPI-NCSC. These large and highly pure populations of neurons can be generated in the absence of cell purification, without genetic manipulation and within a short period of time. Approximately one-third of neurons responded to capsaicin with calcium flux, indicating an active TRPV1 channel. A somewhat larger percentage, 38% of cells, responded to high concentrations of KCl. A panel of 10 marker genes was expressed in all cells. A comparative study with human foetal DRGs showed that based on Brn3a expression and that of other markers, the in vitro generated peptidergic nociceptive sensory neurons showed characteristics of 12 PCW human foetal sensory neurons.

Our results show that manipulating key signalling pathways is essential for in vitro differentiation of hEPI-NCSC into peptidergic nociceptive sensory neurons. In particular, adding SHH and CHIR99021 was essential for up-regulation of NGN2 and RUNX1 expression. CHIR99021 inhibits glycogen synthase kinase-3β (GSK-3β) thereby stabilizing β-catenin and enhancing canonical WNT signalling. BMP signalling was inhibited with LDN193189 to avoid differentiation along the sympathetic neuron lineage. Inhibiting the Notch/Delta pathway, which inhibits NGN1/2 expression, with DAPT was essential to achieve NGN2 expression. There are at least three potential explanations for the cytoplasmic expression of Brn3a in cultured neurons, DRG neurons and boundary caps cells. It could be a stage-specific phenomenon that indicates the onset of Brn3a protein expression. Alternatively there could be a mandatory periodic residency in the cytoplasm to maintain function, similar to SOX10 [35]. Finally, there might be a requirement for a yet to be identified serum factor, or combination of factors. The latter possibility is supported by the fact that in the continued presence of NT-3, BDNF and FBS, BRN3a was predominantly expressed in the nucleus. RUNX1 expression is essential but transient in peptidergic nociceptive sensory neurons, and is thought to be extinguished by HGF/MET action. RUNX1 immunoreactivity declined noticeably after one week of treatment with HGF, but did not completely disappear. It is conceivable that a longer exposure to HGF would have resulted in complete extinction of RUNX1 immunoreactivity.

TRKA was strongly expressed by culture D5 already and immunofluorescence remained intense throughout the culture period, supporting the notion of the peptidergic nociceptive nature of the neurons. TRKA expression early in differentiation of neural crest cells into sensory neurons is well documented (see e.g., George et al, 2010; ref [36]). TRKA expression is then down-regulated in non-peptidergic nociceptive neurons [37]. Intense TRPV1 immunoreactivity was observed already by D5 and it remained strong throughout the culture period. This observation is in agreement with data from Hjerling-Leffler et al [37], who showed in the mouse that capsaicin-sensitive TRPV1 expression is first observed prenatally, at embryonic day 12.5. ISL1 immunoreactivity was also first observed at D5 in our cultures. In contrast, immunoreactivity for SP and CGRP was modest at D5, as expected, and increased with progressing in vitro differentiation.

Spontaneous activity of nociceptive sensory neurons is primarily observed in states of disease, not in healthy peptidergic nociceptive sensory neurons [3]. Substantial spontaneous activity as judged by calcium flux was therefore not expected in hEPI-NCSC-derived peptidergic nociceptive neurons and at 7.8% of monitored cells was indeed infrequent. Cells responding with calcium flux to capsaicin or to high KCl were first observed at culture D+18. Approximately 30% of cells responded to capsaicin with calcium flux both with 100 nM and 1 μM capsaicin, indicating expression of an active TRPV1 channel in a significant subset of cells. While the percentage of responding cells was very similar with the two concentrations, the maximum amplitude was statistically significantly higher with the higher dose, indicating a dose-dependent response. While capsaicin sensitivity is a good indicator of neuronal function, it remains to be determined whether action potentials were generated.

The availability of large and homogenous populations of human peptidergic nociceptive neurons is highly desirable and induced pluripotent stem (iPS) cells are a promising approach to achieve this goal. Chambers et al [38] developed a method for generating iPS lines that consisted of 75% neurons. More than 60% of those expressed TRKA as measured by FACS analysis. Calcium imaging showed that the predominant cell type was, however, P2RX3-positive/TRPV1-negative. Capsaicin (1 μM) induced a response in 1–2% of cells only. Wainger et al [39] used genetic manipulation of mouse fibroblasts to create capsaicin-sensitive nociceptors with 12 genes in viral constructs. Some tetrodotoxin-resistant neurons were obtained as well through re-programming from human fibroblasts from patients with familial dysautonomia; efficiency is, however, unclear. Similarly, Blanchard et al [40] reprogrammed mouse and human fibroblasts by transient (8 days) co-expression of Brn3a and Ngn1 or Brn3a and Ngn2 in doxycycline-inducible lentiviral vectors. Transduced cells expressed markers for the three subtypes of sensory neurons; nociceptive/pruriceptive, mechanoreceptive, and proprioceptive neurons with up to approximately 18% CGRP immunoreactive cells. While the efficiency of reprogramming was low at approximately 4% Brn3a positive cells, of those 20–21% responded to 10 μM capsaicin, indicating that approximately 0.8% of total cells responded to capsaicin. Boisvert et al [41] differentiated human embryonic stem cells into sensory neurons by first inducing the cells to the neuronal cell lineage, subsequently differentiating them into neural crest progenitors and finally exposing the cells to retinoic acid and bone morphogenetic protein-4 (BMP4). When Ngn1 inducible hESC lines were used in which Ngn1 was over-expressed, 63% of cells responded to capsaicin (1 μM). Whether the cells also expressed SP and CGRP was not addressed. In contrast, we show here that all cells were immunoreactive for all three pain-related genes, SP and CGRP and TRPV1. There are many diverse potential reasons for the discrepancy between the 30% of total cells that were TRPV1-sensitive in our study, compared to 63% in NGN1-inducible hESC in the above study [41]. Reasons for the discrepancy are likely complex and could envisioned to be due to differences in gene expression, cell maturation, TRPV1 desensitization, and/or differences in calmodulin, calcineurin or phosphatidylinositol 4,5-bisphosphate (PIP2) signalling. It would be of interest to apply the protocol described by Boisvert et al [41] to hEPI-NCS derived iPS cells. In contrast to other studies, cell sorting was not necessary in our study, and expression of 10 relevant antibody markers was observed in all hEPI-NCSC derived peptidergic nociceptive neurons. The most likely explanation for the straightforward differentiation of hEPI-NCSC into nociceptive peptidergic neurons is their neural crest origin, which is also the embryonic source of nociceptive primary sensory neurons.

We show here that highly pure populations of cells with principal characteristics of peptidergic nociceptive sensory neurons, including response to the TRPV1 agonist capsaicin and expression of SP and CGRP, can be generated from hEPI-NCSC. Because hEPI-NCSC are easily isolated from hairy skin biopsies and can be expanded ex vivo into large populations of multipotent stem cells without the need for cell purification and since they can be differentiated without genetic manipulation, these neurons can be of interest in studies on TRPV1 function, patient-specific disease modelling and drug discovery.

## Conclusions

hEPI-NCSC are a biologically relevant and attractive source to generate human peptidergic nociceptive neurons. They provide a platform for the study of the TRPV1 channel in a patient-specific manner and in an appropriate cellular context. hEPI-NCSC derived peptidergic nociceptive sensory neurons promise to be useful also in drug discovery and disease modelling.

## Supporting information

S1 Table (DOCX)
