# Supplementary material for: Human peptidergic nociceptive sensory neurons generated from human epidermal neural crest stem cells (hEPI-NCSC)
Source: PLoS One. 2018 Jun 28;13(6):e0199996. doi: 10.1371/journal.pone.0199996 (PMC6023242; doi:10.1371/journal.pone.0199996)
Supplement: S1 Table — (DOCX) [file pone.0199996.s001.docx]

**Supplemental Table 1**

**Human peptidergic nociceptive sensory neurons generated from epidermal neural crest stem cells**

Rachel Wilson^1^, Afsara A Ahmmed^1^, Alistair Poll^2^, Motoharu Sakaue^1$^, Alex Laude^3^, Maya Sieber-Blum^1* ¶^

(1) Institute of Genetic Medicine

Centre for Life

Newcastle University

Newcastle upon Tyne

NE1 3BZ

UK

(2) School of Biology

Devonshire Building

Newcastle University

Newcastle upon Tyne
NE1 7RX

UK

(3) The Bio-Imaging Unit

Medical School

Newcastle University

Framlington Place

Newcastle upon Tyne

NE2 4HH

UK

$ **Current address**

Department of Veterinary Medicine

Laboratory of Anatomy II,

School of Veterinary Medicine

Azabu University

1-17-71 Fuchinobe, Chuo-ku

Sagamihara 252-5201

Japan

* **Corresponding Author**

**¶ Current contact details**

Maya Sieber-Blum, Ph.D.

Consultant; USA

E: epincsc@gmail.com

T: +1 262-290-1607

W: [www.mayasieberblum.com](http://www.mayasieberblum.com)

**Equipment**

Class II safety hood (Clean Air, Clean Air Techniek, Woerden, Netherlands)

Confocal microscope (Nikon A1R confocal microscope system, Nikon UK, Kingston Upon Thames, UK)

Digital camera powerShot (A650IS, Canon, Tokyo, Japan)

Dissecting microscope (Stemi 2000, Carl Zeiss UK, Cambridge, UK)

Fluorescence microscope (Imager.Z1, Carl Zeiss UK)

Two-gas incubator at 5% CO_2_ and 5% O_2_ (CO_2_ incubator with O_2_ control, BINDER, Tuttlingen Germany)

Phase bright microscope (Axiovert 40C, Carl Zeiss UK, Cambridge, UK)

**Reagents**

CELLstart CTS (Life Technologies, Cat# A10142-01)

Dulbecco’s phosphate buffer saline (DPBS) with Ca^2+^ and Mg^2+^ (Life Technologies, Cat# 14190-094)

Phosphate buffer saline (PBS) without Ca^2+^ and Mg^2+^ (Life Technologies, Cat# 20012)

Alpha MEM with UltraGlutamine™ I, deoxyribonucleoside and ribonucleosides (Lonza, Basel, Switzerland, Cat# BE02-002F)

NeuroCult-XF basal medium (Stem Cell Technologies, Grenoble, France, Cat# 05760)

NeuroCult-XF Proliferation Supplements (Stem Cell Technologies, Cat# 05763)

Recombinant human epidermal growth factor (rhEGF) (Stem Cell Technologies, Cat# 02653)

Recombinant human fibroblast growth factor 2 (rhFGF2) (R&D Systems, Cat# 233-FB)

0.2% Heparin Sodium Salt Solution in Phosphate Buffer Saline (Stem Cell Technologies, Cat# 07980)

1X SITE+3 liquid medium supplement (Sigma, Poole, UK, Cat# S5295)

Fetal bovine serum (FBS) (HyClone, Thermo Fisher, Cramlington, UK, Cat# SH30070.07)

GlutaMAX-I (Life Technologies, Cat# 35050-038)

Penicillin/streptomycin (Sigma, Cat# P0781)

Matrigel, basement membrane matrix, growth factor reduced (BD Biosciences, Bedford USA, Cat# 354230)

Recombinant human sonic hedgehog (C24II) N-terminus (rhSHH) (R&D Systems, Cat# 1845-SH/CF)

CHIR99021 (Stemgent, Cambridge, MA, Cat# 04-0004)

TrypLE select (Life Technologies, Cat# 12563-011)

Hank’s Balanced Salt Solution with Ca^2+^ and Mg^2+^ (Life technologies, Cat#14025)

Neurocult NS-A Basal Medium (Stem Cell Technologies, Cat# 05750)

Neurocult NS-A Proliferation Supplements (Stem Cell Technologies, Cat# 05753)

Recombinant human Stem Cell Factor protein (rhSCF) (R&D Systems, Cat# 255-SC/CF)

Recombinant human brain-derived neurotrophic factor (rhBDNF) (R&D Systems, Abingdon, UK, Cat# 248-BD/CF)

Recombinant human neurotrophin 3 (rhNT3) (R&D Systems, Cat# 267-N3/CF)

Recombinant human β-nerve growth factor (rhβNGF) (R&D Systems, Cat# 256-GF/CF)

2-mercaptoethanol (2ME) (Sigma, Cat# M3148)

DAPT (Tocris Bioscience, Cat# 2634)

Stemolecule^TM^ LDN193189 (Stemgent, Cat# 04-0074)

Neurocult NS-A Differentiation Supplements (Stem Cell technologies, Cat# 05754)

B27 supplement minus vitamin A (Life Technologies, Paisley, UK, Cat # 12587-010)

Ascorbic acid (Sigma, Cat# A4544)

Paraformaldehyde (PFA) (Sigma, Cat# 158127)

dbcAMP monophosphate sodium salt (Sigma, Cat# D0260)

Recombinant human Hepatocyte Growth Factor (rhHGF) (R&D Systems, Cat# 296-HGN)

Vectashield hard set mounting medium with 4’,6-diamidino-2-phenylindole (DAPI) (Vector Laboratories, Peterborough, UK, Cat# H-1500)

Normal goat serum (NGS) (Sigma, Cat# G9023)

Triton X-100 (Sigma, Cat# T8787)

Dimethyl Sulfoxide (DMSO) (Sigma, Cat# D4540)

0.22µM Syringe Filters (Pall, Cat# 4612)

35-mm culture plate (Fisher Scientific UK, Leicestershire, UK**,** Cat# 430165)

150-mm plates (VWR, Cat# 353025)

13-mm in diameter round cover glasses (VWR, Leicestershire, UK, Cat# 631-0149)

Nunc^TM^ 4-well dishes (Thermscientific, Cat# 144444)

**List of Primary Antibodies**

Mouse anti-**Neurogenin-1** antibody (1:7.4) (Abcam, Cambridge, UK, Cat# ab89461)

Sheep anti-**Calcitonin Gene Related peptide (CGRP)** antibody (1:800) (Enzo life sciences, Cat# BML-CA1137-0100)

Rabbit anti-**Neurogenin-2** antibody (1:1000) (Abcam, Cat# 154293)

Mouse anti-**Substance P** antibody (1:1000) (Abcam, Cat# ab14184)

Rabbit anti-**RUNX1** antibody (1:50) (Abcam, Cat# ab23980)

Mouse anti-**TRKA** antibody (1:200) (Abcam, Cat# ab86474)

Rabbit anti-**BRN3A** antibody (1:200) (Millipore, Cat# MAB5945)

Mouse anti-**β III tubulin** antibody **(**1:200) (Millipore, Cat# MAB1637)

Rabbit anti-**TRPV1** antibody (1:1000) (Neuromics, Cat# RA14113)

Mouse anti-**Islet 1** antibody (1:200) (Hybridoma Bank, Cat# 39.4D5)

**List of Secondary antibodies***

*All of secondary antibodies were purchased from Stratech Scientific, Newmarket, UK

Alexafluor-594 conjugated donkey anti-mouse IgG (Cat# 715-585-150)

Alexafluor-488 conjugated donkey anti-sheep IgG (Cat# 713-545-147)

Alexafluor-488 conjugated goat anti-rabbit IgG (Cat# 111-545-144)

Dylight-594 conjugated goat anti-mouse IgG (Cat# 115-515-146)
